# Primary care management of the coronavirus (COVID-19)

**DOI:** 10.4102/safp.v62i1.5115

**Published:** 2020-03-31

**Authors:** Bob Mash

**Affiliations:** 1 Division of Family Medicine and Primary Care, Faculty of Medicine and Health Sciences, University of Stellenbosch, Cape Town, South Africa

**Keywords:** coronavirus, COVID-19, SARS-CoV-2, primary care, continuing medical education, clinical management

## Abstract

South Africa is in the grip of a novel coronavirus pandemic (COVID-19). Primary care providers are in the frontline. COVID-19 is spread primarily by respiratory droplets contaminating surfaces and hands that then transmit the virus to another person’s respiratory system. The incubation period is 2–9 days and the majority of cases are mild. The most common symptoms are fever, cough and shortness of breath. Older people and those with cardiopulmonary co-morbidities or immunological deficiency will be more at risk of severe disease. If people meet the case definition, the primary care provider should immediately adopt infection prevention and control measures. Diagnosis is made by a RT-PCR test using respiratory secretions, usually nasopharyngeal and oropharyngeal swabs. Mild cases can be managed at home with self-isolation, symptomatic treatment and follow-up if the disease worsens. Contact tracing is very important. Observed case fatality is between 0.5% and 4%, but may be overestimated as mild cases are not always counted. Primary care providers must give clear, accurate and consistent messages on infection prevention and control in communities and homes.

## Introduction

Severe acute respiratory syndrome coronavirus 2 (SARS-CoV-2) was first detected in December 2019.^1^ As I write this, the coronavirus disease 2019 (COVID-19) pandemic is hitting South Africa and events are unfolding fast as new measures are taken on a daily basis to contain its spread within communities. This article does not attempt to keep up with the latest announcements and news, but to share what we know about the virus and its clinical management. The article is targeted at primary care providers, general practitioners and family physicians.

It is clear that containing the virus will require an all-of-society response and that the pandemic will impact multiple sectors such as health, business, tourism, education, and trade. Our response is intended to contain the spread of the virus and slow the growth of the epidemic in order to allow the health system to cope.

There is a risk of stigma for those infected and a plethora of misinformation, panic and anxiety.

Facilities and healthcare practices should adequately prepare themselves to cope with the epidemic, and a checklist, along with other resources, has been provided on the National Institute of Communicable Diseases (NICD) website at http://www.nicd.ac.za/diseases-a-z-index/COVID-19/COVID-19-resources/.

## Clinical picture

The incubation period appears to be 2–9 days, with a median of 5 days. The four most common symptoms are^2,3^:

- Fever (83%)
- Cough (82%)
- Difficulty breathing (31%)
- Fatigue and myalgia (11%)

The most common presentation is myalgia, malaise, cough, and a low-grade fever. Difficulty breathing may develop in the second week (average 8 days) due to viral pneumonia.

Symptoms such as sore throat, runny nose, headache, chest pain, confusion, diarrhoea, nausea and vomiting are also possible, but much less common. Most patients (90%) have more than one symptom, but only 15% have fever, cough and shortness of breath together. The cough is most often a dry cough.

Laboratory findings are not that helpful. The most consistent finding is a lymphopaenia, while C-reactive protein is unreliable. Abnormalities are seen on X-ray in about 60% of patients and bilateral pneumonia is the commonest finding.^2^ The most consistent finding is a ground glass appearance due to interstitial infiltrates.

It should be emphasised that the majority of people will have a mild self-limiting illness (81%), while 14% may be more severe with hypoxia and dyspnea. Around 5% may become critically ill with respiratory failure, septic shock or multi-organ dysfunction.^4^

Co-infection with another virus is uncommon and secondary bacterial infection is also less common than with influenza.

Patients should be treated in a separate room, although not necessarily an airborne infection isolation room, as measures to prevent contact and surface contamination are most important.

## Case definition

The current case definition for a suspected case or person under investigation is^5^:

- Person with **acute respiratory illness** (sudden onset of at least one of the following: cough, sore throat, shortness of breath or fever irrespective of severity)

This in combination with:

- At least one of the following epidemiological criteria in the **14 days prior** to onset of symptoms:
  ■ Was in close contact with a confirmed or probable case of SARS-Cov-2 infection.
  ■ Had a history of travel to areas with presumed ongoing community transmission of SARS-CoV-2.
  ■ Worked in, or attended, a healthcare facility where patients with SARS-CoV-2 infections were being treated.

Anyone admitted with a severe pneumonia of unknown aetiology should also be regarded as a possible case.

## Diagnosis

The main diagnostic is a real-time reverse transcriptase polymerase chain reaction (RT-PCR) test on respiratory secretions. Secretions are typically obtained by a nasopharyngeal (Box 1) and oropharyngeal swab (Box 2) placed in universal transport medium and kept cool (between 2 °C – 8 °C). The test can also be performed on sputum or tracheal aspirate if available. In hospital, bronchoalveolar lavage can also be used to collect a sample. Serological testing on acute and convalescent serum samples can confirm infection, but is not useful for immediate diagnosis.

BOX 1 Collecting a nasopharyngeal swab.^6^

- Use only nylon or rayon flocked swabs with perforated, flexible plastic shaft. Cotton-tipped, calcium alginate swabs or swabs with wooden shafts should not be used as residues present in these materials may inhibit PCR assays.
- Gently insert nasopharyngeal flocked swab into the nostril aiming backwards, along the floor of the nasal cavity, until the nasopharynx is reached. Be careful not to insert swab upwards. If resistance is encountered during insertion of the swab, remove it and try the other nostril. The distance from the nose to the ear gives an estimate of the distance the swab should be inserted.
- Gently rotate the swab and hold in place for a few seconds.
- Slowly withdraw swab.
- Unscrew and remove the cap from the tube with transport medium.
- Insert the swab directly into a vial containing universal transport medium (UTM).
- Break plastic shaft at the break point so that it can fit in the UTM tube.
- Close the tube with the lid.
- Refrigerate at 2 °C – 8 °C.

*Source:* National Institute for Communicable Diseases. Guidelines for case-finding, diagnosis, management and public health response in South Africa [homepage on the Internet]. 2020 [cited 2020 Mar 18]. Available from: http://www.nicd.ac.za/wp-content/uploads/2020/02/Guidelines-for-case-finding-diagnosis-management-and-public-health-response-in-South-Africa.pdf

PCR, polymerase chain reaction.

BOX 2 Collecting an oropharyngeal swab.^6^

- Keeping the same pair of gloves on, and holding the universal transport medium (UTM) with the nasopharyngeal swab in, take a second flocked swab and open it at the plastic shaft.
- Ask the patient to tilt their head back and open mouth wide.
- Hold the tongue down with a tongue depressor.
- Have the patient say ‘aahh’ to elevate the uvula.
- Swab each tonsil first, then the posterior pharynx in a ‘figure 8’ movement.
- Avoid swabbing the soft palate and do not touch the tongue with the swab tip as this procedure can induce the gag reflex.
- Insert the swab directly into the same UTM vial containing the nasopharyngeal swab.
- Break plastic shaft at the break point so that it can fit in the UTM tube.
- Close the tube with the lid.
- Refrigerate at 2 °C – 8 °C.

*Source:* National Institute for Communicable Diseases. Guidelines for case-finding, diagnosis, management and public health response in South Africa [homepage on the Internet]. 2020 [cited 2020 Mar 18]. Available from: http://www.nicd.ac.za/wp-content/uploads/2020/02/Guidelines-for-case-finding-diagnosis-management-and-public-health-response-in-South-Africa.pdf

All cases are notifiable as per the NICD: http://www.nicd.ac.za/notifiable-medical-conditions/.

## Case management

If someone meets the case definition given above then infection prevention and control measures should be immediately adopted and specimens taken for a diagnosis. Mild disease can be managed at home (Box 3) with symptomatic treatment and self-isolation (Box 4).

BOX 3 Criteria for mild disease in adults and adolescents.^7^

SpO_2_ ≥ 95%

Respiratory rate < 25/min

Heart rate < 120/min

Temperature 36 °C – 39 °C

Mental state normal

*Source:* National Institute for Communicable Disease. Clinical management of suspected or confirmed COVID-19 disease [homepage on the Internet]. 2020 [cited 2020 Mar 18]. Available from: http://www.nicd.ac.za/wp-content/uploads/2020/03/Clinical_management_of_suspected_or_acute_COVID_V1.1_13.03.20_updated.pdf

Box 4 Instructions for self-isolation.^7^

- Patients should stay in a specific room and use their own bathroom (if possible). Patients should avoid unnecessary travel and unnecessary contact with other people.
- Where contact is unavoidable, the patient should wear a surgical mask, and maintain a distance of at least 1 m (preferably 2 m) from other people.
- Patients should clean their hands frequently with soap and water. Alcohol-based sanitisers may also be used, provided they contain at least 70% alcohol.
- Patients should practise good cough and sneeze hygiene, by using a tissue, and then immediately discarding the tissue in a lined trash can, followed by washing hands immediately.
- Patients should not have visitors in their home. Only those who live in their home should be allowed to stay.
- If they live in shared accommodation (university halls of residence or similar) with a communal kitchen, bathroom and living area, they should stay in their room with the door closed, only coming out when necessary, wearing a face mask if they do so.
- Patients should avoid sharing household items such as dishes, cups, eating utensils and towels. After using any of these, the items should be thoroughly washed with soap and hot water.
- All high-touch surfaces such as table tops, counters, toilets, phones, computers, etc. should be cleaned both appropriately and frequently.
- If patients need to wash laundry at home before the results are available, then they should wash all laundry at the highest temperature compatible for the fabric using laundry detergent. This should be above 60 °C. If possible, they should tumble dry and iron using the highest setting compatible with the fabric. Disposable gloves and a plastic apron should be used when handling soiled materials, if possible, and all surfaces, as well as the area around the washing machine, should be cleaned. Laundry should not be taken to a laundrette. The patient should wash their hands thoroughly with soap and water after handling dirty laundry (remove gloves first if used).
- Patients should know who to call if they develop any worsening symptoms, so that they can safely be reassessed, as 10% – 15% of patients may worsen. Older patients (> 50 years) or those with co-morbidities are most likely to worsen.
- Provide a patient information sheet.

*Source:* National Institute for Communicable Disease. Clinical management of suspected or confirmed COVID-19 disease [homepage on the Internet]. 2020 [cited 2020 Mar 18]. Available from: http://www.nicd.ac.za/wp-content/uploads/2020/03/Clinical_management_of_suspected_or_acute_COVID_V1.1_13.03.20_updated.pdf

Self-isolation can be ended when the patient’s symptoms have improved or resolved and they have two consecutive negative RT-PCR tests at least 24–48 hours apart.

If severe or critically ill, take or send them to an emergency or resuscitation area, keep waste in a designated area and do not share equipment with other patients. Consult a more senior doctor.

There is no specific treatment for COVID-19 and treatment is supportive.^8^ Treat fever and myalgia with paracetamol and offer oxygen if there is difficulty breathing.

Differential diagnosis would include other viral pneumonia, such as from seasonal influenza, bacterial pneumonia (conventional and atypical organisms) and, in the HIV immunocompromised population, *Pneumocystis jirovecii* pneumonia.

Critically ill patients may need intubation and ventilation and treatment in a high care or intensive care unit.

## Contact tracing

Once someone is diagnosed with COVID-19, contact tracing must start immediately. Contacts are divided into two groups:

- Close contacts: A person having had face-to-face contact (≤ 2 m) or was in a closed environment with a COVID-19 case; this includes, amongst others, all persons living in the same household as a COVID-19 case, and people working closely in the same environment as a case. A healthcare worker or other person providing direct care for a COVID-19 case, while not wearing recommended personal protective equipment (PPE) (e.g. gowns, gloves, NIOSH-certified disposable N95 respirator, eye protection). A contact in an aircraft sitting within two seats (in any direction) of the COVID-19 case, travel companions or persons providing care, and crew members serving in the section of the aircraft where the index case was seated.
- Casual contacts: Anyone not meeting the definition for a close contact, but with possible exposure.

Close contacts should self-quarantine at home and be monitored by daily telephonic follow up for 14 days for the development of symptoms. Casual contacts should ensure social distancing and avoid non-essential contact and self-monitor for 14 days. If symptoms develop, they should be investigated as already described.

## Case fatality rates

Observed case fatality rates may be higher than reality as it is difficult to count all of the mild cases making up the denominator. Most observations place the overall case fatality rate as less than 3% (range 0.5% – 4%) and modelling suggests between 0.3% and 1.0%. This is probably more than seasonal influenza, on a par with swine flu or measles, and considerably less than Spanish flu, severe acute respiratory syndrome (SARS) or Middle East respiratory syndrome (MERS).

Figures from China suggest that case fatality increases with age from 0.2% in young adults to 3.6% (60–69 years), 8% (70–79 years) and 15% (≥ 80 years). In China, the overall mortality for males (2.8%) was higher than females (1.7%).^4^

People with cardiopulmonary co-morbidity (e.g. cardiovascular disease, diabetes, structural lung damage, tuberculosis or chronic obstructive pulmonary disease) have a higher morbidity and mortality. In South Africa we can postulate that immunocompromised patients, particularly those with poorly controlled HIV, will be at higher risk, as might tobacco smokers.

## Spread of the infection

It is estimated that an infected person on average infects between 2 and 4 other people. This is on a par with the common cold, and far less than chickenpox or measles. Infection is mostly by respiratory droplets contaminating surfaces and being passed by hand to one’s own respiratory tract. Infection is less common by airborne spread of droplets that are breathed in, although some hospital procedures may generate an aerosol (e.g. Bilevel Positive Airway Pressure machines).

Transmission is most likely when the person is symptomatic. Transmission may be possible between asymptomatic people in the incubation period, but the extent of this is not known. Viral shedding can be detected for 1–4 weeks after symptoms resolve, but the extent of infectivity is not known as particles detected may not be viable.

## Infection prevention and control

When handling a suspected or confirmed case you should isolate them in a single room or separate them from other patients by at least 2 m and put a surgical mask on the patient. All staff providing clinical care should wear a surgical mask, apron and gloves.

If you are going to perform a procedure that creates an aerosol then wear a fit-tested N95 respirator during the procedure instead of a surgical mask and eye protection as well as the apron and gloves. Such procedures include taking nasopharyngeal or oropharyngeal swabs, sputum samples or tracheal aspirates. The single room should be adequately ventilated.

Medical equipment, including stethoscope or blood pressure cuffs and thermometers should be cleaned and disinfected before using on another patient.

In the community, people should avoid infection by:

- Regularly washing hands for 20 seconds with soap and water or hand sanitiser – particularly after visiting the bathroom, before and after eating, after blowing your nose, or coughing or sneezing.
- Avoiding touching the face or nose with uncleaned hands.
- Avoid close contact with sick people, stay at least 2 m away from someone that is coughing.

People should avoid spreading the infection:

- Social distancing, staying at home and avoiding contact with groups of people.
- Self-isolation if you are a contact of a case, a person under investigation or a confirmed case.
- Cough or sneeze into the crook of your elbow or into a tissue, which is disposed of immediately in a closed container. Wash hands.
- Do not share with other people items such as clothing, bedding, towels, cell phones, uncovered food, books and magazines.
- Close the lid when you flush the toilet.
- Wipe frequently touched areas regularly with a disinfectant.
- Hand hygiene.
- It is not useful to wear a surgical mask unless you are a suspected or confirmed case.

## Self-care and team-based care

The COVID-19 epidemic may put even more pressure on healthcare workers who already feel overstretched. Doctors and nurses who are caring for people with COVID-19 will also be anxious about infecting themselves and taking the infection home to their families. The epidemic may be with us for several months and we will need to pace ourselves. Mindful awareness of levels of stress and one’s need to take a break is important in order to recharge during the day. We are not superhuman and also need to ensure sufficient sleep and food to continue working effectively. Self-compassion and compassion for those around us in our teams is important. Acknowledging and witnessing one another’s experiences may help the team to cope with what can feel unreal. If necessary, make use of psychological support services.

## References

[1] Phan LT, Nguyen TV, Luong QC, et al Importation and human-to-human transmission of a novel coronavirus in Vietnam. N Engl J Med. 2020;382(9):872–874. 10.1056/NEJMc2001272 31991079 PMC7121428

[2] Chen N, Zhou M, Dong X, et al Epidemiological and clinical characteristics of 99 cases of 2019 novel coronavirus pneumonia in Wuhan, China: A descriptive study. Lancet. 2020;395(10223):507–513. 10.1016/S0140-6736(20)30211-7 32007143 PMC7135076

[3] Huang C, Wang Y, Li X, et al Clinical features of patients infected with 2019 novel coronavirus in Wuhan, China. Lancet. 2020;395(10223):497–506. 10.1016/S0140-6736(20)30183-5 31986264 PMC7159299

[4] Novel Coronavirus Pneumonia Emergency Response Epidemiology Team [The epidemiological characteristics of an outbreak of 2019 novel coronavirus diseases (COVID-19) in China]. Zhonghua Liu Xing Bing Xue Za Zhi. 2020;41(2):145–151. 10.3760/cma.j.issn.0254-6450.2020.02.003 32064853

[5] National Insititute for Communicable Diseases 2019-nCoV quick reference for health workers [homepage on the Internet]. 2020 [cited 2020 Mar 17]. Available from: http://www.nicd.ac.za/wp-content/uploads/2020/02/2019-nCov-Quick-reference-v3-03.02.2020-final.pdf

[6] National Institute for Communicable Diseases Guidelines for case-finding, diagnosis, management and public health response in South Africa [homepage on the Internet]. 2020 [cited 2020 Mar 18]. Available from: http://www.nicd.ac.za/wp-content/uploads/2020/02/Guidelines-for-case-finding-diagnosis-management-and-public-health-response-in-South-Africa.pdf

[7] National Institute for Communicable Disease Clinical management of suspected or confirmed COVID-19 disease [homepage on the Internet]. 2020 [cited 2020 Mar 18]. Available from: http://www.nicd.ac.za/wp-content/uploads/2020/03/Clinical_management_of_suspected_or_acute_COVID_V1.1_13.03.20_updated.pdf

[8] WHO Clinical management of severe acute respiratory infection when novel coronavirus (nCoV) infection is suspected [homepage on the Internet]. 2020 [cited 2020 Mar 17]. Available from: https://www.who.int/publications-detail/clinical-management-of-severe-acute-respiratory-infection-when-novel-coronavirus-(ncov)-infection-is-suspected

